# Visit-to-visit variability in estimated glomerular filtration rate predicts hospitalization and death due to cardiovascular events

**DOI:** 10.1007/s10157-019-01695-9

**Published:** 2019-01-28

**Authors:** Akira Suzuki, Yoshitsugu Obi, Terumasa Hayashi, Naoto Kotani, Yukari Uemura, Enyu Imai, Hirofumi Makino, Akira Hishida

**Affiliations:** 1 Department of Internal Medicine, Japan Community Healthcare Organization Osaka Hospital, 4-2-78 Fukushima Fukushima-ku, Osaka, 553-0003 Japan; 2 Harold Simmons Center for Kidney Disease Research and Epidemiology, University of California, Irvine, CA USA; 3 Department of Kidney Disease and Hypertension, Osaka General Medical Center, Osaka, Japan; 4 Pharmaceuticals and Medical Devices Agency, Center for Product Evaluation, Biostatistics Group, Tokyo, Japan; 5 Clinical Research Support Center, Biostatistics Division, The University of Tokyo Hospital, Tokyo, Japan; 6 Nakayamadera Imai Clinic, Takarazuka, Hyogo Japan; 7 Okayama University, Okayama, Okayama Japan; 8 Yaizu City Hospital, Yaizu, Shizuoka Japan

**Keywords:** Chronic kidney disease, Variability, Estimated glomerular filtration rate, Cardiovascular mortality, End-stage kidney disease

## Abstract

**Background:**

Greater variability in estimated glomerular filtration rate (eGFR) is associated with mortality in patients with chronic kidney disease (CKD). However, the association between eGFR variability and cardiovascular (CV) mortality and/or end-stage kidney disease (ESKD) in the CKD population is not very clear. This study aimed to clarify whether such an association exists.

**Methods:**

We analyzed a final cohort of 2869 eligible Asian patients with CKD. Patients were stratified into three groups according to eGFR variability during the first year and were followed-up for a median of 3.15 years. Primary CV composite endpoints were hospitalization or death due to CV events, and renal composite endpoints were doubling of serum creatinine levels or ESKD. Multivariate Cox hazard models adjusted for classical risk factors and eGFR slope were used to examine the CV and renal risk associated with eGFR variability.

**Results:**

CV endpoints were observed in 14 (2.89%), 25 (5.69%), and 41 (10.79%) patients and renal endpoints were observed in 165 (27.6%), 235 (39.0%), and 298 patients (50.9%) in the lowest, intermediate, and highest tertiles of eGFR variability, respectively. Patients in the highest tertile were at a significantly higher risk for CV events (hazard ratio 1.90; 95% confidence interval 1.03–3.71) than those in the lowest tertile. However, there was no association between eGFR variability and renal endpoints.

**Conclusions:**

Variability in eGFR can predict CV outcomes among patients with CKD.

## Introduction

Chronic kidney disease (CKD) is a huge public health problem that affects more than 10% of the population worldwide [1] and approximately 13.3 million in Japan [2]. Impaired kidney function is associated with incident cardiovascular disease (CVD), all-cause mortality, and end-stage kidney disease (ESKD) [3–5]. The number of patients with ESKD on chronic dialysis has continued to increase worldwide over the past few decades, and ESKD has thus emerged as a financial burden. A single estimated glomerular filtration rate (eGFR) was historically adopted as a predictive variable for ESKD in observational studies; however, recent reports have demonstrated that a decreased rate of eGFR was an independent predictor for ESKD in patients with CKD [6, 7]. Notably, a transient increase in eGFR was also associated with risks for CVD and all-cause mortality [8]. These findings suggest that eGFR variation may be a novel predictive factor for poorer clinical outcomes of patients with CKD. Two retrospective observational studies revealed that eGFR variability was a risk factor for CVD and all-cause mortality [9, 10]. The aim of the present study was to prospectively investigate the relationship between eGFR variability, and CVD and ESKD incidence among the CKD population.

## Materials and methods

### Study population

The design and method used for the Chronic Kidney Disease Japan Cohort (CKD-JAC) study have been published elsewhere [11]. Briefly, the inclusion criteria were as follows: (1) Japanese or Asian patients living in Japan; (2) age between 20 and 75 years; and (3) a broad spectrum of CKD stages, defined as an eGFR of 10–59 ml/min/1.73 m^2^. The eGFR was calculated using the estimation equation for Japanese patients [12]. The exclusion criteria were as follows: (1) polycystic kidney disease, human immunodeficiency virus infection, liver cirrhosis, active cancer, or cancer treatment within the past 2 years; (2) history of transplant and chronic dialysis; (3) pregnancy in women; and (4) refusal to provide informed consent. Recruitment started in April 2007, and 2,966 participants were followed-up until March 2013. The median follow-up period was 3.9 years. The protocol was approved by the Ethics Committee of each participating medical institute, and all participants provided informed consent. Baseline characteristics of all patients enrolled in CKD-JAC have been described previously [13].

### Predictor variable

The predictor variable was the eGFR variability during the first year of the study. The variability was defined as the absolute residual of the eGFR regression line/the expected eGFR estimated by the linear regression line at each time point. The variability for each patient was calculated as the mean value of the variability. Patients were stratified into three groups according to the tertile of the mean variability during the first year.

### Primary endpoints

Two primary composite endpoints were defined: (1) hospitalization due to congestive heart failure or death due to CVD and (2) doubling of serum creatinine levels or ESKD. CVD included acute myocardial infarction, heart failure, arrhythmia, stroke, and aorta dissection. The doubling of serum creatinine levels was defined as three consecutive values of serum creatinine that were twice as high as the baseline serum creatinine.

### Statistical analysis

Continuous variables were presented as mean and standard deviation or median and range, and categorical variables were presented as frequency and percentage. For missing values, we used the multiple imputation method. Differences in baseline characteristics were tested using one-way analysis of variance for continuous variables, and the Chi square test was used for categorical variables.

To test the association between time to the endpoint and the mean variability in eGFR, the Kaplan–Meier curve and Cox proportional hazard model were used for unadjusted and multivariate-adjusted analysis, respectively. Baseline covariates included in the models were age, sex, body mass index, systolic blood pressure, diastolic blood pressure, prior CVD, diabetes mellitus, use of diuretics, use of antihypertensive agents, and baseline eGFR. The variability in eGFR was included in models two and three. The eGFR slope was included in model three. *p* < 0.05 was considered statistically significant. All analyses were performed using SAS software version 9.4.

## Results

**Fig. 1:**
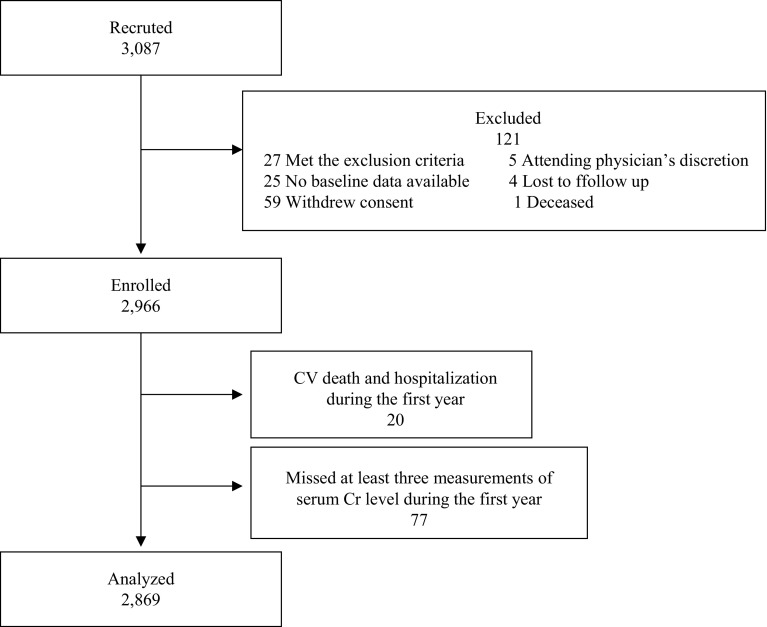
Patients disposition. *CV* cardiovascular. *Cr* creatinine

A total of 2,966 patients with stage 3–5 CKD were enrolled (Fig. 1). Twenty patients were excluded due to CVD or death occurring during the first year of observation. Seventy-seven patients were excluded because their serum creatinine levels were measured two times or less during the first year. Therefore, a total of 2869 patients were eligible and were followed-up for a median of 3.15 years (0.05–4.34 years). The eligible subjects were stratified into three groups according to the eGFR variability during the first year of the study period. Baseline characteristics stratified by the eGFR variability are shown in Tables 1 and 2. The tertile with the highest eGFR variability showed significantly more instances of ischemic heart disease, stroke, and diabetes. This tertile also demonstrated significantly older age, lower diastolic blood pressure, higher serum uric acid levels, lower eGFR, lower serum albumin levels, and lower hemoglobin levels.

**Table 1:** Baseline demographic characteristics of the tertile of eGFR variability

	Lowest (*n* = 956)		Intermediate (*n* = 957)		Highest (*n* = 956)		*p* value
	*n*	(%)	*n*	(%)	*n*	(%)	
Gender (male)	598	62.6	603	63.0	585	61.2	0.6952
Ischemic haeart disease	99	10.4	120	12.5	177	18.5	< 0.0001
Stroke	76	8.0	111	11.6	140	14.6	< 0.0001
Aorta disease or PAD	60	6.3	71	7.4	95	9.9	0.0098
Diabetes mellitus	216	22.6	314	32.8	426	44.6	< 0.0001
Use of diuretics	176	18.41	267	27.9	427	44.67	< 0.0001
Use of antihypertensive drug	780	81.6	779	81.4	797	83.37	0.4642

*PAD* peripheral artery disease

**Table 2:** Baseline characteristics of the tertile of eGFR variability

	Lowest (*n* = 956)				Intermediate (*n* = 957)				Highest (*n* = 956)				*p* value
	Average	Median	S.D.	(Min–Max)	Average	Median	S.D.	Min–Max	Average	Median	S.D.	(Min–Max)	
Age	58.9	61.0	11.9	(21.0–77.0)	60.7	63.0	11.5	(24.0–76.0)	61.9	64.0	11.0	(22.0–77.0)	< 0.0001
BMI (kg/m^2^)	23.4	23.2	3.7	(12.8–39.8)	23.5	23.0	3.7	(10.3–36.7)	23.6	23.2	3.9	(11.7–38.7)	0.4854
Systoric BP (mmHg)	130.6	130.0	18.0	(80.0–235.0)	131.9	131.0	17.8	(73.0–202.0)	132.5	131.3	19.6	(67.7–207.7)	0.0725
Diastoric BP (mmHg)	77.7	78.0	11.3	(40.0–127.7)	76.4	76.3	11.4	(34.5–117.0)	74.7	74.7	12.4	(32.7–127.3)	< 0.0001
Heart rate (bpm)	75.3	74.0	12.6	(44.0–122.0)	75.4	74.0	12.5	(43.0–120.0)	76.6	75.0	13.3	(46.0–126.0)	0.0904
Uric asid ( mg/dL	7.0	6.9	1.4	(3.0–12.1)	7.1	7.1	1.6	(1.2–13.4)	7.4	7.2	1.7	(2.2–14.2)	< 0.0001
Serum creatinine (mg/dL)	2.0	1.7	1.0	(0.8–6.2)	2.2	1.8	1.1	(0.8–8.5)	2.2	2.0	1.1	(0.8–7.8)	0.0002
BUN (mg/dL)	28.2	25.4	13.0	(5.1–107.2)	31.2	28.0	13.9	(4.0–91.0)	34.8	31.0	15.8	(3.1–144.4)	< 0.0001
Serum Na (mEq/L)	140.7	141.0	3.0	(124.0–191.0)	140.4	140.0	2.9	(123.0–183.0)	140.2	140.0	2.7	(120.0–150.0)	0.0031
Serum K (mEq/L)	4.6	4.6	0.5	(3.1–6.4)	4.7	4.6	0.6	(2.5–7.0)	4.7	4.6	0.6	(2.1–7.2)	0.1217
Serum CL (mEq/L)	106.4	106.5	3.1	(94.0–116.0)	106.6	107.0	3.6	(88.0–122.0)	106.3	106.0	4.2	(77.0–119.0)	0.1995
Serum P (mg/dL)	3.4	3.4	0.7	(1.6–7.1)	3.5	3.4	0.7	(1.8–8.6)	3.7	3.6	0.7	(1.7–7.3)	< 0.0001
Serum Ca (mg/dL)	9.1	9.1	0.5	(5.4–10.7)	9.0	9.0	0.5	(6.4–11.6)	8.9	9.0	0.6	(5.9–10.6)	< 0.0001
Serum Albumin (g/dL)	4.1	4.1	0.4	(2.1–5.3)	4.0	4.0	0.4	(1.4–5.1)	3.9	3.9	0.5	(1.7–5.3)	< 0.0001
Hemoglobin (g/dL)	12.6	12.4	1.8	(7.1–18.5)	12.1	11.9	1.8	(5.2–18.5)	11.6	11.5	1.8	(6.0–19.7)	< 0.0001
Urinary protein/creatinine (g/gCr)	1.0	0.6	1.3	(0.0–8.7)	1.7	0.8	5.7	(0.0–97.6)	1.8	0.8	3.4	(0.0–41.2)	0.0167
eGFR(ml/min/1.73 m^2^)	30.4	30.3	12.1	(6.5–61.9)	28.7	28.5	12.1	(6.4–72.6)	27.4	25.8	12.2	(5.4–74.4)	< 0.0001

*BMI* body mass index, *BP* blood pressure, *BUN* blood urea nitogen

**Fig. 2:**
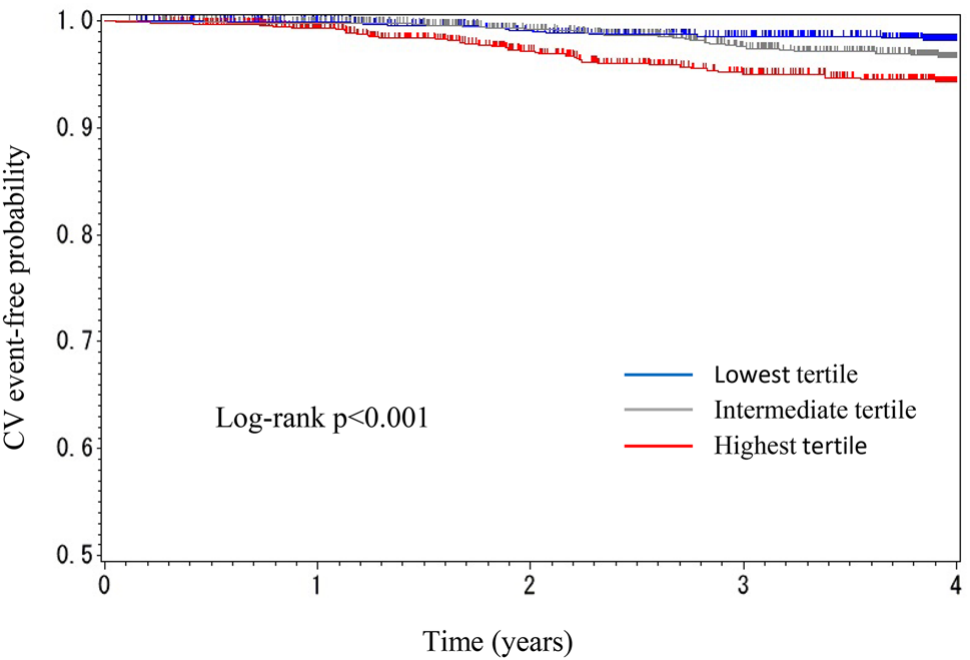
Kaplan–Meier curve (time to CV event) by tertile of eGFR variability

During follow-up, 14 cardiovascular (CV) events (2.89%) occurred in the lowest tertile, 25 (5.69%) occurred in the intermediate tertile, and 41 (10.79%) occurred in the highest tertile of variability. Kaplan–Meier survival curves for CV events are shown in Fig. 2. The highest tertile showed significantly worse CV event-free survival compared to the lowest tertile (*p* < 0.0001; log rank test).

A multivariate-adjusted Cox proportional hazard model indicated that the patients in the highest tertile showed an increased risk of CV events compared with those in the lowest tertile [hazard ratio (HR), 1.90; 95% confidence interval (CI), 1.03–3.71] (Table 3). A significantly increased risk was also associated with the following variables: age (HR 1.06; 95% CI 1.02–1.09); history of CV event (HR 2.53; 95% CI 1.55–4.08); male sex (HR 1.86; 95% CI 1.10–3.28); use of diuretics (HR 1.73; 95% CI 1.06–2.85); and baseline eGFR (HR 0.97; 95% CI 0.95–0.99).

**Table 3:** Multivariate-adjusted Cox proportional hazard for CV events

	H.R.	95% C.I.	*p* value
Model 1			
Gender (male vs female)	1.85	[1.10, 3.26]	0.0260
Age (1-year increase)	1.06	[1.03, 1.09]	0.0008
BMI	1.02	[0.95, 1.09]	0.6018
History of heart disease	2.60	[1.60, 4.19]	< 0.0001
Diabetes Mellitus	1.20	[0.72, 2.00]	0.4953
Diastoric BP	0.98	[0.96, 1.01]	0.1334
Systoric BP	1.01	[1.00, 1.03]	0.1536
Use of diuretics	1.86	[1.14, 3.05]	0.0127
Use of ACE	0.94	[0.57, 1.51]	0.7970
eGFR (1ml/min/1.73 m^2^ increse)	0.97	[0.95, 0.99]	0.0032
Model 2			
eGFR variability (intermediate vs lowest)	1.41	[0.74, 2.80]	0.3083
(Highest vs lowest)	1.92	[1.04, 3.75]	0.0429
Gender (male vs female)	1.86	[1.11, 3.29]	0.0243
Age (1-year increase)	1.06	[1.02, 1.09]	0.0009
BMI	1.02	[0.95, 1.09]	0.6266
History of heart disease	2.52	[1.55, 4.06]	0.0002
Diabeyes Mellitus	1.14	[0.68, 1.91]	0.6105
Diastoric BP	0.98	[0.96, 1.01]	0.1748
Systoric BP	1.01	[1.00, 1.03]	0.1727
Use of diuretics	1.72	[1.05, 2.83]	0.0321
Use of ACE	0.90	[0.54, 1.45]	0.6735
eGFR (1 ml/min/1.73 m^2^ increse)	0.97	[0.95, 0.99]	0.0038
Model 3			
eGFR variability (Intermediate vs lowest)	1.41	[0.74, 2.80]	0.3089
(Highest vs lowest)	1.90	[1.03, 3.71]	0.0469
Gender (male vs female)	1.86	[1.10, 3.28]	0.0248
Age(1-year increase)	1.06	[1.02, 1.09]	0.0009
BMI	1.02	[0.95, 1.09]	0.6296
History of heart disease	2.53	[1.55, 4.08]	0.0002
Diabetes	1.12	[0.67, 1.89]	0.6603
Diastoric BP	0.98	[0.96, 1.01]	0.1736
Systoric BP	1.01	[0.99, 1.03]	0.1988
Use of diuretics	1.73	[1.06, 2.85]	0.0296
Use of ACE	0.90	[0.54, 1.45]	0.6757
eGFR (1 ml/min/1.73 m^2^ increse)	0.97	[0.95, 0.99]	0.0037
eGFR slope (/year)	0.99	[0.94, 1.03]	0.5468

*H.R*. Hazard ratio, *C.I*. Confidence interval, *BMI* body mass index, *BP* blood pressure, *ACE* angiotensin converting enzyme

**Fig. 3:**
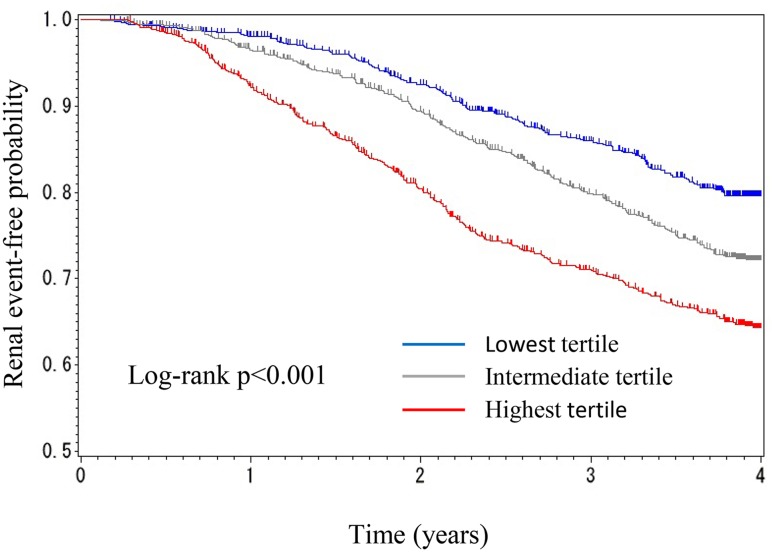
Kaplan–Meier curve (time to ESKD or doubling of serum creatinine) by tertile of estimated glomerular filtration rate variability

However, 165 renal events (27.6%) occurred in the lowest tertile, 235 (39.0%) occurred in the intermediate tertile, and 298 (50.9%) occurred in the highest tertile of variability. Although the Kaplan–Meier survival curve for renal events revealed a significant association between the eGFR variability and the time for renal events (Fig. 3), the association was not observed after adjustment for the eGFR slope in model three (Table 4).

**Table 4:** Multivariate-adjusted Cox proportional hazard models for renal events

	H.R.	95% C.I.	*p* value
Model 1			
Gender (male vs female)	2.00	[1.68, 2.39]	< 0.0001
Age (1-year increase)	0.98	[0.97, 0.99]	< 0.0001
BMI	1.02	[1.00, 1.04]	0.1225
History of heart disease	0.84	[0.66, 1.04]	0.1206
Diabetes mellitus	1.38	[1.15, 1.66]	0.0005
Diastoric BP	0.98	[0.97, 0.99]	0.0001
Systoric BP	1.03	[1.02, 1.03]	< 0.0001
Use of diuretics	1.11	[0.93, 1.33]	0.2377
Use of ACE	0.82	[0.68, 0.97]	0.0234
eGFR (1 ml/min/1.73 m^2^ increse)	0.90	[0.89, 0.91]	< 0.0001
Model 2			
eGFR variability (intermediate vs lowest)	1.21	[0.98, 1.49]	0.0762
(Highest vs lowest)	1.66	[1.35, 2.04]	< 0.0001
Gender (male vs female)	2.02	[1.70, 2.41]	< 0.0001
Age (1-year increase)	0.98	[0.97, 0.99]	< 0.0001
BMI	1.02	[1.00, 1.04]	0.0823
History of heart disease	0.81	[0.64, 1.01]	0.0687
Diabetes Mellitus	1.29	[1.07, 1.55]	0.0072
Diastoric BP	0.98	[0.97, 0.99]	0.0002
Systoric BP	1.03	[1.02, 1.03]	< 0.0001
Use of diuretics	1.05	[0.88, 1.26]	0.5778
Use of ACE	0.79	[0.66, 0.94]	0.0095
eGFR (1 ml/min/1.73 m^2^ increse)	0.90	[0.89, 0.91]	< 0.0001
Model 3			
eGFR variability (intermediate vs lowest)	1.16	[0.94, 1.43]	0.1761
(Highest vs lowest)	1.19	[0.97, 1.48]	0.1057
Gender (male vs female)	1.91	[1.59, 2.29]	< 0.0001
Age (1-year increase)	0.98	[0.97, 0.98]	< 0.0001
BMI	1.02	[0.99, 1.04]	0.1999
History of heart disease	0.75	[0.60, 0.95]	0.0166
Diabetes Mellitus	1.21	[0.99, 1.46]	0.0580
Diastoric BP	0.98	[0.97, 0.99]	< 0.0001
Systoric BP	1.02	[1.02, 1.03]	< 0.0001
Use of diuretics	1.21	[1.01, 1.45]	0.0338
Use of ACE	0.75	[0.63, 0.90]	0.0020
eGFR (1 ml/min/1.73 m^2^ increse)	0.89	[0.88, 0.89]	< 0.0001
eGFR slope (/year)	0.84	[0.83, 0.86]	< 0.0001

*H.R*. Hazard ratio, *C.I*. Confidence nterval, *BMI* body mass index, *BP* blood pressure, *ACE* angiotensin converting enzyme

## Discussion

The CKD-JAC study was a multicenter, prospective cohort study that recruited pre-dialysis CKD Japanese patients. In this setting, variability in eGFR was associated with an increased risk of CV hospitalization or death, even after adjustment for classical risk factors and a decreased rate of eGFR. Patients in the highest tertile of variability demonstrated a 90% increased risk compared with those in the lowest tertile, whereas baseline eGFR only had a modest association and the decreased rate of eGFR had no significant association with CV events. CKD is one of the established risk factors for CVD. Therefore, eGFR variability may provide more powerful prognostic information for CVD than baseline eGFR and eGFR slope.

Although the variation in eGFR can be generated by hemodynamic changes, the risk of variability in eGFR was independent of blood pressure and use of antihypertensive drugs or diuretics. The highest variability tertile comprised patients with a history of ischemic heart disease, stroke, and diabetes; however, the association between CV risk and variability in eGFR was significant after adjustment for these comorbidities. The highest tertile also demonstrated older age, lower diastolic blood pressure, higher serum uric acid levels, higher serum creatinine levels, higher blood urea nitrogen levels, higher serum Na levels, higher serum phosphate levels, lower serum Ca levels, lower serum albumin levels, lower Hb levels, and higher urinary protein levels. It was not possible to adjust the multivariate analysis by urinary protein due to the many missing values (deficit rate 67.4%).

Two recent retrospective cohort studies revealed that the variability in eGFR was independently associated with all-cause mortality for CKD patients [9, 10]. They defined the variability as the coefficient of variation of the regression line coefficient of eGFR, or as the absolute value of the residual of the eGFR regression line. The definition of variability in eGFR in the present study was the absolute residual of the eGFR regression line/the expected eGFR estimated by the linear regression line at each time point, which was modified from the definition indicated by Perkins. This parameter should be more accurate because it is independent of the eGFR value. Further studies are needed to evaluate the predictive value of each formula because there is no accepted definition of variability in eGFR. Community-acquired acute kidney injury (AKI), which was excluded from the previous studies, was included in the present study. Exclusion of AKI may have compromised the results because it has been recognized as a risk factor for long-term renal outcome and mortality.

In the setting of outpatient practice, visit-to-visit differences are commonly observed not only in the laboratory results but also in physical parameters such as blood pressure, heart rate, and body weight. The individual variation in eGFR may be affected by the volume status, blood pressure, deterioration (amelioration) of comorbidities, and modified medications. Of note, variations in blood pressure have been shown to be associated with not only the incidence of CVD and mortality [14], but also the decreased rate of renal function [15]. Recently, blood pressure variability was reported to be associated with the progression of carotid arteriosclerosis [16]. These findings could explain the results in the present study.

Variability in eGFR can be attributed to the loss of renal autoregulation of GFR depending on both the loss of functioning renal mass and atherosclerosis of renal arteries. We also analyzed the association between variability in eGFR and the composite renal outcome (ESKD or doubling of serum creatinine). Variability in eGFR was significantly and independently associated with the composite renal outcome in the multivariate-adjusted Cox proportional hazard model without the eGFR slope; however, it demonstrated no significant association in the analysis adjusted with the eGFR slope. These findings suggest that variability in eGFR may be caused predominantly by atherosclerosis rather than loss of renal mass because elevation of eGFR was not caused by the restoration of functioning nephrons in patients with CKD stage 3–5, which was getting progressively worse in most cases. In other words, patients with transient elevations in eGFR in the course of observation may have more severe systemic atherosclerotic changes in comparison to that in patients without transient elevation of eGFR.

The present study had some limitations. First, the cohort included only Asian patients; therefore, the findings may not be generalizable to other populations. Second, all patients were followed by nephrologists at established regional medical institutions. It is impossible for all Japanese patients to be treated in the same setting due to the limited number of nephrologists. A distinct number of CKD patients are supposed to be followed by cardiologists, diabetologists, and general physicians. Therefore, the findings of the present study may not be applicable to those patients. Third, the changes in prescribed medications, including diuretics and antihypertensives, were not recorded during the observational period. Modifying the doses of these drugs can affect the eGFR via volume status and blood pressure, which would result in greater variability in eGFR. Forth, urinary protein is a well-established risk factor for CV event and renal outcome; however, it was impossible to add urinary protein into the multivariate-adjusted analysis because of the many missing values (deficit rate 67.4%).

## Conclusion

In conclusion, the present study suggests that variability in eGFR is an independent predictor of hospitalization or death due to CV events among patients with CKD stage 3–5. Further study should be conducted to clarify the pathological basis linking the variability of eGFR to CV events.
